# The Myositis Overlap Conundrum: Differentiating Polymyositis from Inclusion Body Myositis

**DOI:** 10.14789/ejmj.JMJ25-0061-CR

**Published:** 2026-02-10

**Authors:** MOE HEIN, WIN LAE LAE AUNG, EAINDRA MYAT THU, ABM ASHRAFUL HUDA

**Affiliations:** 1 Department of Rheumatology, Hull University Teaching Hospital, Hull, UK; 1 Department of Rheumatology, Hull University Teaching Hospital, Hull, UK

**Keywords:** polymyositis, inclusion body myositis, autoimmune myopathy, myositis overlap, dysphagia

## Abstract

We describe the case of a 77-year-old woman who presented with progressive weakness in both lower extremities, dysphagia to both solids and liquids, and severe weight loss. Her recent experience with statin-induced rhabdomyolysis was initially considered to be associated with her high creatine kinase (CK) level. Nevertheless, she became weaker and experienced dysphagia even after the cancellation of statin therapy. Lab tests indicated that there were constantly high CK (3347 U/L) and positive anti-PM-Scl-100 antibody, which indicated an autoimmune inflammatory myopathy. Lower limb MRI revealed diffuse myositis, and muscle biopsy revealed active myopathy with inflammation and rimmed vacuoles, characteristic of polymyositis/inclusion body myositis (IBM). She received high-dose corticosteroids and then intravenous immunoglobulin (IVIg) and methotrexate as treatment for polymyositis. Although she experienced an improvement in her limb weakness, she was still dysphagic. The case demonstrates that the diagnosis of inflammatory myopathies in older people is complex, and there is a need to identify inclusion body myositis early in the course to inform proper management.

## Introduction

Idiopathic inflammatory myopathies (IIMs) are a heterogeneous family of autoimmune diseases of skeletal muscle characterised by chronic inflammation, elevated serum muscle enzymes, and progressive muscle weakness^1)^. The primary subtypes are polymyositis (PM), dermatomyositis (DM), and inclusion body myositis (IBM). These are distinguished on clinical grounds, histopathology, autoantibody status, and response to treatment^2)^.

Polymyositis is a rare variant, accounting for 5% of all IIMs^3)^. It is typically characterised by symmetric proximal muscle weakness, elevated creatine kinase (CK), and usually responds to corticosteroids and other immunosuppressive therapy. In comparison, inclusion body myositis is the most common acquired myopathy in adults over 50 years of age. It is a clinically characteristic disorder with slowly progressive, asymmetric weakness, especially in the quadriceps and the flexors of the fingers, and histologically, endomysial inflammation, rimmed vacuoles, and intracytoplasmic inclusions^4, 5)^.

Muscle biopsy is the primary diagnostic tool, in which inflammatory alterations, necrosis, and the presence of typical rimmed vacuoles in IBM are directly measurable by histopathological methods, which are essential for differentiating it from other inflammatory myopathies^1, 4, 5)^.

The separation of PM and IBM may be difficult in elderly patients, as both may have similar clinical and laboratory manifestations. Detection of myositis- related autoantibodies (anti-PM-Scl-100 or anti-cN1A) may further complicate the diagnostic process. In contrast to polymyositis, IBM is usually resistant to immunosuppressive therapy, and misdiagnosis may lead to unsuitable long-term immunosuppression and delay in the provision of supportive care^6)^.

This case is presented to highlight the diagnostic complexity of distinguishing polymyositis from inclusion body myositis in an elderly patient with overlapping clinical, serological, and pathological findings. It also highlights the value of a multidisciplinary approach to diagnosis, including imaging, electrophysiology, serology, and, especially, muscle biopsy, to achieve diagnostic accuracy. The most crucial lesson from this case is that IBM should be considered in older patients with poor steroid response to the disease, even when autoimmune antibodies indicate polymyositis, and that a muscle biopsy is crucial for making the correct diagnosis and determining management.

### Case presentation

A 77-year-old female patient was brought to the same-day emergency with a history of generalised malaise, bilateral lower limb weakness, and dysphagia to both solids and liquids requiring five weeks. She also complained of unintentional weight loss of 2 stones (about 12 kg) during this time. Her medical history included hypertension and hypercholesterolaemia. A month ago, she was hospitalised due to rhabdomyolysis, which was suspected to be caused by statin therapy (CK 4590 U/L), and statin was withdrawn.

### Neurological examination findings

In the current presentation, neurological assessment showed the presence of proximal lower limb weakness (Medical Research Council [MRC] 4/5) and normal distal strength (5/5), intact reflexes, and sensation. Speech and language therapy (SALT) assessment confirmed oropharyngeal dysphagia. The detailed neurological findings are summarised in Table 1.

**Table 1:** Neurological examination findings

Examination parameter	Right side	Left side
Tone	Normal; no spasticity or rigidity	Normal; no spasticity or rigidity
Power-upper limb (proximal)	Normal (5/5)	Normal (5/5)
Power-upper limb (distal)	Normal (5/5)	Normal (5/5)
Power-lower limb (proximal)	Hip flexion, extension, abduction, adduction: 4/5	Hip flexion, extension, abduction, adduction: 4/5 (slightly weaker)
Power-lower limb (distal)	Knee flexion/extension and ankle dorsiflexion/plantarflexion: 5/5	Knee flexion/extension and ankle dorsiflexion/plantarflexion: 5/5
Reflexes	Regular and symmetrical; no clonus	Regular and symmetrical; no clonus
Sensation	Normal to all modalities	Normal to all modalities
Proprioception	Normal	Normal
Fasciculations	None observed	None observed
Coordination	Normal in upper limbs; unable to assess lower limbs due to weakness	Normal in the upper limbs; unable to determine the lower limbs due to weakness
Gait	Not tested (unable to stand due to weakness)	Not tested (unable to stand due to weakness)
Cranial nerves	Normal; no tongue fasciculations	Normal; no tongue fasciculations

### Laboratory investigations

Laboratory investigations are summarised in Table 2, showing markedly elevated creatine kinase with regular inflammatory markers and negative viral and vasculitis screening.

**Table 2:** Laboratory investigations

Test	Result	Reference range/interpretation
Haemoglobin	135 g/L	115-155 g/L (female)
White blood cell count	9.24 × 10^9^/L	4.0-11.0 × 10^9^/L
Serum creatine kinase (CK)	3347 U/L	< 200 U/L
C-reactive protein (CRP)	2 mg/L	< 5 mg/L
Hepatitis B surface antigen	Non-reactive	Non-reactive
Hepatitis C antibody	Non-reactive	Non-reactive
HIV-1/2 antibody	Non-reactive	Non-reactive
Cytomegalovirus (CMV) IgM	Non-reactive	Non-reactive
Epstein-Barr virus (EBV) IgM	Non-reactive	Non-reactive
Vasculitis screen (ANCA, ANA, ENA)	Normal	No autoantibodies detected

### Autoantibody profile

Autoimmune serology revealed a positive anti-PM-Scl-100 antibody, which is associated with polymyositis, dermatomyositis, or systemic sclerosis overlap syndromes. All other myositis- and connective tissue disease-related antibodies were negative. However, testing for anti-cN1A (cytosolic 5′- nucleotidase 1A) antibody, a biomarker associated with inclusion body myositis, yielded an equivocal result, warranting repeat testing for confirmation. The detailed antibody profile is summarised in Table 3.

**Table 3:** Myositis antibody profile

Test	Result	Reference range/interpretation
Myositis antibody profile	Positive	Indicates one or more myositis-associated antibodies detected
Anti-EJ Ab (immunoblot)	Negative	< 20 U/mL = Negative
Anti-Jo-1 Ab (immunoblot)	Negative	< 20 U/mL = Negative
Anti-Ku Ab (immunoblot)	Negative	< 20 U/mL = Negative
Anti-MDA5 Ab (immunoblot)	Negative	< 20 U/mL = Negative
Anti-Mi-2a Ab (immunoblot)	Negative	< 20 U/mL = Negative
Anti-Mi-2b Ab (immunoblot)	Negative	< 20 U/mL = Negative
NXP-2 Ab (immunoblot)	Negative	< 20 U/mL = Negative
Anti-OJ Ab (immunoblot)	Negative	< 20 U/mL = Negative
Anti-PL-12 Ab (immunoblot)	Negative	< 20 U/mL = Negative
Anti-PL-7 Ab (immunoblot)	Negative	< 20 U/mL = Negative
Anti-PM-Scl-100 Ab (immunoblot)	Positive	≥ 20 U/mL = Positive; associated with PM/DM/systemic sclerosis overlap
Anti-PM-Scl-75 Ab (immunoblot)	Negative	< 20 U/mL = Negative
Ro-52 Ab serum	Negative	< 20 U/mL = Negative
SAE-1 Ab (immunoblot)	Negative	< 20 U/mL = Negative
Anti-SRP Ab (immunoblot)	Negative	< 20 U/mL = Negative
TIF-1γ Ab (immunoblot)	Negative	< 20 U/mL = Negative
Anti-HMGCR Ab	Negative	< 20 U/mL = Negative
Anti-cN1A Ab (cytosolic 5′-nucleotidase 1A)	Equivocal	20-25 U/mL = Equivocal; biomarker associated with inclusion body myositis

### Radiological/Imaging findings

Magnetic resonance imaging (MRI) of the brain was unremarkable. MRI of the lower limbs demonstrated diffuse increased T2 signal intensity involving the pelvic, thigh, and calf muscles, more pronounced on the left, consistent with diffuse myositis. Computed tomography (CT) of the thorax, abdomen, and pelvis showed no evidence of malignancy; however, a 33 × 16 mm lesion in the right hepatic lobe was identified and subsequently characterised as benign on triphasic CT imaging.

### Electromyography (EMG)

Electromyography showed that insertional activity was more evident with fibrillation potentials across a variety of muscle types, including the right anterior tibialis, gastrocnemius, vastus lateralis, and biceps. Motor unit action potentials (MUAPs) were short-duration, polyphasic, and early-recruited, which do not exclude a myopathic process. Table 4 summarises the detailed EMG findings of each of the tested muscles.

**Table 4:** Electromyography (EMG) summary

Muscle	Nerve	Roots	Insertional activity (IA)	Fibrillation (Fib)	Positive sharp waves (PSW)	Fasciculations (Fasc)	Amplitude (Amp)	Duration (Dur)	Polyphasia (Polyp)
Right tibialis anterior	Deep peroneal (fibular)	L4-L5	CRD	1+	1+	None	N	N	2+
Right gastrocnemius (medial head)	Tibial (popliteal fossa)	S1-S2	CRD	2+	2+	None	N	1-	1+
Right vastus lateralis	Femoral	L2-L4	1+	1+	1+	None	N	1-	2+
Right deltoid (posterior)	Axillary	C5-C6	1+	1+	1+	None	N	1-	2+
Right biceps brachii	Musculocutaneous	C5-C6	1+	1+	1+	None	N	1-	2+
Right first dorsal interosseous	Ulnar	C8-T1	N	None	None	None	N	N	N

### Muscle biopsy

**Figure 1:**
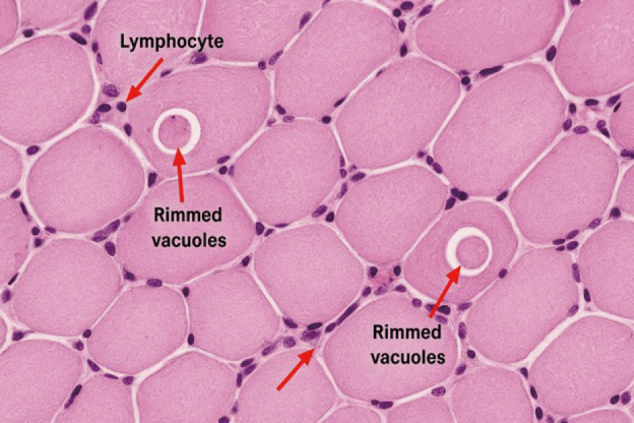
Muscle biopsy, haematoxylin and eosin (H&E) stain, × 200 magnification — showing endomysial inflammation with scattered lymphocytic infiltrates (arrow) and rimmed vacuoles within muscle fibres.

The muscle biopsy, under histopathological examination, showed active myopathic changes with endomysial inflammation and scattered lymphocytic infiltrates containing rimmed vacuoles, indicative of inclusion body myositis (Figure 1).

**Figure 2:**
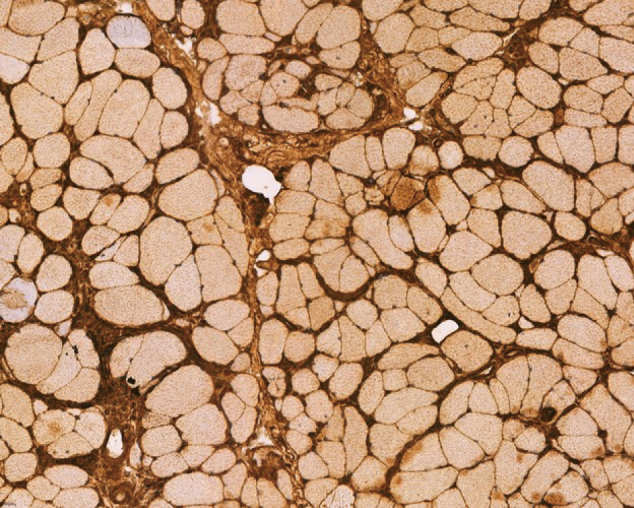
Muscle biopsy, MHC class I immunohistochemistry, × 200 magnification — demonstrating diffuse upregulation of sarcolemmal MHC class I staining (arrow), consistent with inflammatory myopathy.

MHC class I immunohistochemical stains revealed widespread upregulation of sarcolemmal expression, a characteristic of an inflammatory myopathy (Figure 2).

**Figure 3:**
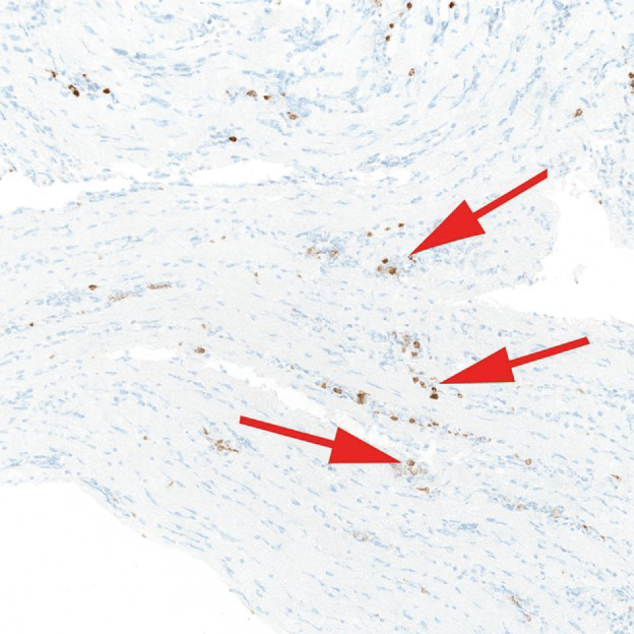
Muscle biopsy, CD3 immunohistochemistry, × 100 magnification — highlighting numerous CD3-positive T lymphocytes infiltrating endomysial connective tissue (arrow).

Subsequent staining for CD3 revealed numerous CD3+ T lymphocytes infiltrating the endomysial connective tissue, supporting T-cell-mediated inflammation (Figure 3).

**Figure 4:**
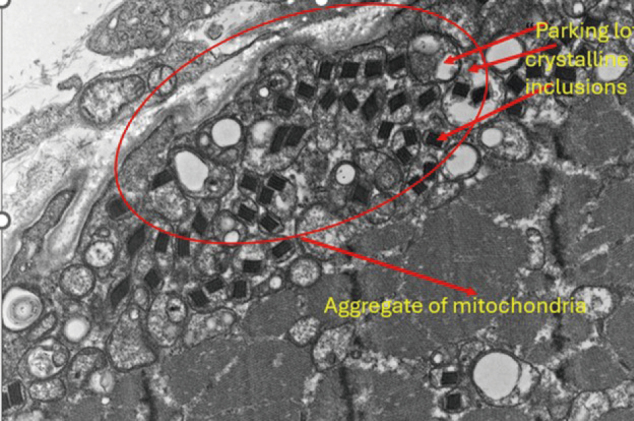
Electron microscopy of muscle tissue, × 25,000 magnification — showing aggregates of mitochondria and "parking-lot" crystalline inclusions (arrow), supporting inclusion body myositis.

Under electron microscopy, the biopsy showed aggregates of mitochondria and peculiar crystalline inclusions resembling parking lots, which contribute to the diagnosis of inclusion body myositis (Figure 4).

Even though p62 staining failed to demonstrate ubiquitinated material, the rimmed vacuoles, endomysial inflammation, and the typical ultrastructural inclusions, in combination with the clinical presentation, were powerful indicators of an inclusion body myositis diagnosis. It was also reported that early-stage biopsies do not always reflect all the classic pathological features, further adding to diagnostic uncertainty.

### Management and clinical course

The patient was treated as having a presumptive diagnosis of polymyositis and started to receive intravenous methylprednisolone (500 mg every day for five days), then oral prednisolone (60 mg every day). Given the persistently elevated CK and poor response, intravenous immunoglobulin (IVIg) was administered, and methotrexate was added as an adjunctive immunosuppressant. IVIg was repeated after four weeks.

A slight increase in proximal limb strength was monitored over several weeks. Still, dysphagia remained, and there was a need to continue enteral nutrition through a nasogastric tube with the continuous assistance of the speech and language therapy team. The follow-up was to be multidisciplinary to track disease progression and reassess therapeutic response, with repeat antibody testing and imaging to further diagnose inclusion body myositis.

## Discussion

Polymyositis is a rare form of idiopathic inflammatory myopathy, which constitutes about 5% of all cases^1)^. It is characterised by autoimmune-mediated inflammation of muscle tissue, in which CD8+ T cells aggressively target muscle fibres that have abnormally expressed MHC class I molecules, leading to myofiber necrosis and weakness^2)^. Diagnosis is based on a combination of clinical evidence, elevated serum creatine kinase (CK), electromyographic abnormalities, and typical changes on muscle biopsy^3^^-^^5)^. The primary treatment remains corticosteroids, often combined with immunosuppressive agents such as methotrexate and azathioprine; those who do not respond may receive intravenous immunoglobulin (IVIg) or newer immunomodulatory drugs^7)^.

On the other hand, inclusion body myositis (IBM) is the most prevalent acquired myopathy in individuals above 50 years of age^8)^. It is a degenerative and inflammatory skeletal muscle disorder, often accompanied by cytosolic 51A (cN1A) autoantibodies^9)^. The pathogenesis encompasses endomysial invasion by CD8+ T cells and macrophages, alongside degenerative alterations, including protein aggregation and mitochondrial dysfunction^10)^. IBM clinically appears with progressive, but slowly progressive, asymmetrical weakness, especially of the quadriceps and finger flexors. In contrast to polymyositis, IBM is usually resistant to immunosuppressive treatment. Histopathological changes also include endomysial inflammation, rimmed vacuoles, and inclusion bodies^11)^.

In this case, polymyositis and IBM overlapped to a significant degree, complicating the initial diagnosis. The presence of anti-PM-Scl-100 antibodies and MRI paradigms initially supported a diagnosis of polymyositis. Nevertheless, the patient's advanced age, limited response to corticosteroids, and rimmed vacuoles on muscle biopsy were more indicative of IBM.

This conclusion was corroborated by further pathological examination. Electron microscopy revealed the typical parking-lot crystalline inclusions, which are not characteristic of polymyositis and support a diagnosis of IBM^8)^. Even though the p62 staining was negative, this could also be a sampling variation, and the staining has been repeated to confirm. The rimmed vacuoles observed in the digital review were clear and unlikely to be artefacts.

Myositis autoantibody panel was a further complication. Although anti-PM-Scl-100 antibodies were evidently positive, indicating the possibility of an overlap syndrome, anti-cN1A antibody tests were inconclusive. These antibodies are now accepted as a biomarker for IBM, and it has been suggested that repeat testing in 6 months be performed to facilitate diagnosis^12)^.

Currently, the patient is being treated with suspected polymyositis in the form of corticosteroids, IVIg, and methotrexate. However, with the existing clinical, histological, and serological evidence, inclusion body myositis is the most probable diagnosis. To validate this, an MRI repeat and, perhaps, a repeat muscle biopsy are under consideration.

A clear distinction between polymyositis and IBM is essential, as their management and prognosis vary significantly. Polymyositis is generally responsive to immunosuppression, whereas IBM is not and requires a supportive, multidisciplinary approach that includes physiotherapy, speech and swallowing therapy, and nutritional management^13)^. Identifying IBM at an early stage can reduce unnecessary prolonged steroid use and maximise patient quality of life through individualised supportive care.

## Conclusions

Inclusion body myositis (IBM) must be considered in elderly patients with progressive muscle weakness and dysphagia when there are serological indications of polymyositis. Diagnostic confirmation is necessary and can be achieved through a muscle biopsy, which helps differentiate IBM from other inflammatory myopathies.

Here, even with early intervention using corticosteroids, intravenous immunoglobulin, and methotrexate because the patient had presumed polymyositis, limb strength improved modestly, yet the patient continued to have dysphagia; the results are more compatible with IBM. She was finally discharged with a percutaneous endoscopic gastrostomy (PEG) tube to feed herself, and she walks with the help of a Zimmer frame.

Follow-up assessment is scheduled at six months, with magnetic resonance imaging (MRI) of the lower limbs repeated, myositis antibodies repeated, and a possible repeat muscle biopsy to clarify the diagnosis further and assess disease progression. Early recognition of IBM is essential to avoid unnecessary, protracted immunosuppressive treatment and to initiate timely, supportive multidisciplinary treatment, such as physiotherapy, nutrition, and speech and swallowing therapy, to maximise quality of life.

## Data availability

Not applicable. No datasets were generated or analyzed during this case report.

## Author contributions

MH conceived and designed the study and supervised the overall project. WLLA contributed to patient data collection and clinical interpretation. EMT assisted with data acquisition and literature review. ABMH supported clinical analysis and manuscript drafting. MH drafted the manuscript, coordinated revisions, and served as the corresponding author. All authors read and approved the final manuscript.

## Conflicts of interest statement

The authors declare that there are no conflicts of interest.
